# Disruptive Technologies for Environment and Health Research: An Overview of Artificial Intelligence, Blockchain, and Internet of Things

**DOI:** 10.3390/ijerph16203847

**Published:** 2019-10-11

**Authors:** Frederico M. Bublitz, Arlene Oetomo, Kirti S. Sahu, Amethyst Kuang, Laura X. Fadrique, Pedro E. Velmovitsky, Raphael M. Nobrega, Plinio P. Morita

**Affiliations:** 1 School of Public Health and Health Systems, University of Waterloo, Waterloo, ON N2L 3G1, Canada; fred.bublitz@uwaterloo.ca (F.M.B.); arlene.oetomo@uwaterloo.ca (A.O.); kirti.sahu@uwaterloo.ca (K.S.S.); amethyst.kuang@uwaterloo.ca (A.K.); lxavierfadrique@uwaterloo.ca (L.X.F.); pevelmovitsky@uwaterloo.ca (P.E.V.); raphael.nobrega@uwaterloo.ca (R.M.N.); 2 Center for Strategic Technologies in Health (NUTES), State University of Paraiba (UEPB), Campina Grande, PB 58429-500, Brazil; 3 Institute of Health Policy, Management, and Evaluation, University of Toronto, Toronto, ON M5T 3M6, Canada; 4 Research Institute for Aging, University of Waterloo, Waterloo, ON N2J 0E2, Canada; 5 Department of Systems Design Engineering, University of Waterloo, Waterloo, ON N2L 3G1, Canada; 6 eHealth Innovation, Techna Institute, University Health Network, Toronto, ON M5G 2C4, Canada

**Keywords:** environment, global health, surveillance system, climate change, disruptive technologies, IoT, blockchain, artificial intelligence, reference architecture

## Abstract

The purpose of this descriptive research paper is to initiate discussions on the use of innovative technologies and their potential to support the research and development of pan-Canadian monitoring and surveillance activities associated with environmental impacts on health and within the health system. Its primary aim is to provide a review of disruptive technologies and their current uses in the environment and in healthcare. Drawing on extensive experience in population-level surveillance through the use of technology, knowledge from prior projects in the field, and conducting a review of the technologies, this paper is meant to serve as the initial steps toward a better understanding of the research area. In doing so, we hope to be able to better assess which technologies might best be leveraged to advance this unique intersection of health and environment. This paper first outlines the current use of technologies at the intersection of public health and the environment, in particular, Artificial Intelligence (AI), Blockchain, and the Internet of Things (IoT). The paper provides a description for each of these technologies, along with a summary of their current applications, and a description of the challenges one might face with adopting them. Thereafter, a high-level reference architecture, that addresses the challenges of the described technologies and could potentially be incorporated into the pan-Canadian surveillance system, is conceived and presented.

## 1. Introduction

The technological evolution of Information and Communication Technology has simplified and optimized the collection of health and environmental data [1]. For example, the increasing storage capacities of cloud servers now allow portable sensors to continuously collect data. However, the collected data have not always been utilized to their fullest potential. Nonetheless, inexpensive data storage and the increased computational capacity of technologies are driving the creation of large datasets that contain health and environmental data with untapped potential [2].

The University of Waterloo provided recommendations to the Climate Change and Innovation Bureau (CCIB) of Health Canada on the potential to incorporate innovative technologies in the scoping and development of pan-Canadian monitoring and surveillance activities associated with environmental impacts on health and the healthcare system [3]. These activities include integrating data from multiple health and environmental data ecosystems into a platform that enables researchers, policy makers, and health officials to use analytic services for generating insights from the data available; as well as the operationalization of the use of these data within the aforementioned agencies and in the community, providing a centralized place for the storage, processing, and analysis of health and environmental data [4].

The implementation of these activities may benefit from several disruptive technologies such as: (a) cloud storage and processing systems, (b) Artificial Intelligence (AI) including Machine Learning (ML), (c) data science, (d) Blockchain, (e) Internet of Things (IoT), (f) mobile health, (g) wearables, and (h) Ambient Assisted Living. These disruptive technologies have widespread use in multiple domains dealing with Big Data, such as influenza surveillance and air quality monitoring, thus showing great promise in prospectively supporting the implementation of the pan-Canadian surveillance system [5,6,7,8,9,10,11,12].

This paper comes at a crucial time. Since our climate is rapidly changing and impacting human health and well-being, we must also adapt to the needs of our healthcare systems. “The greatest health threat of the 21st century” is climate change and it is an enormous challenge that requires inventive minds and various resources to protect human health [13]. As identified by the Intergovernmental Panel on Climate Change (IPCC), climate change will have impacts on health resulting from:

- increased morbidity and mortality due to increased atmospheric temperatures causing heat-related illnesses such as heat stroke, acute cardiovascular disease, and renal disease;
- increased morbidity and mortality due to reduced air quality as a result of greenhouse gases (GHGs), which causes health issues such as ischemic heart disease, stroke, and lung cancer;
- increased prevalence of vector-borne diseases due to warmer temperatures causing an expansion of geographic range of insects and other species; and
- increased frequency and intensity of extreme weather events, such as floods, droughts, and hurricanes. These will cause a chain reaction affecting food security, housing, and infrastructure, resulting in lost income for those affected by these events [14].

Canada has seen a dramatic increase in the costs of extreme weather events such as extreme temperatures, floods, and wildfires [15,16]. These changes are harmful to the health of Canadians and, according the Insurance Bureau of Canada, the costs of these extreme weather events has increased steadily from $100 million per year 2 decades ago to $2 billion per year in 2013–2014 [17].

Protecting the environment is a critical step in protecting public health, and creative interventions are required to find cost-effective methods that address budget limitations [18]. This paper explores the current work that is being done using disruptive technologies to solve some of the challenges (as seen in the use case sections). The Lancet Countdown 2018 report states that the current impacts of climate change, exposures, and vulnerabilities present an unacceptably high risk to human health, stressing the importance of addressing these areas [19]. A pan-Canadian surveillance system incorporating some elements of the technologies described in this paper will be beneficial for the surveillance and monitoring activities by public health practitioners and policy makers to make informed decisions to protect the health and well-being of individuals [20].

The Ubiquitous Health Technology Lab (UbiLab) at the University of Waterloo was contracted by CCIB of Health Canada to provide an assessment of the aforementioned technologies. In this paper, we present a narrative review of the existing literature and the use of these technologies in environmental and health monitoring scenarios (individual remote monitoring and population-level surveillance) as well as a discussion of the possible challenges that require consideration in the implementation of these proposed components of the pan-Canadian surveillance system. The goal of this research initiative is to provide some examples of actual and potential uses of such technologies in the areas of environmental and health-related monitoring. Additionally, a potential software architecture specifically for environment and health that incorporates such technologies and that could be considered by the CCIB in developing its pan-Canadian monitoring and surveillance activities is a corresponding goal.

## 2. Methods

The project was conducted using descriptive research and follows a traditional narrative literature review.

The UbiLab team conducted three rounds of internal team discussions exploring the most relevant papers and technologies. During the discussions, a brainstorming session was held to support the development of use cases that could integrate AI, Blockchain, and IoT. Lastly, the UbiLab prepared a conceptual architecture that considers the way these innovative technologies could be adapted and integrated to support the scoping and development of monitoring and surveillance activities.

Although this was not a scoping systematic review, we followed recommendations made by Levac et al. [21] as our aim is to be more descriptive. In this methodological framework, six stages are proposed for the scoping review studies: (1) identifying the research question; (2) identifying relevant studies; (3) study selection; (4) charting the data; (5) collating, summarizing, and reporting the results; and (6) consultation (optional).

### 2.1. Identifying the Research Question and Relevant Studies

For this review, our focus was to understand “*how disruptive technologies, which focuses on AI, IoT, and Blockchain, can help to improve Environment and Health Research?*” A literature review, conducted via the Google search engine, included searching for relevant grey literature (including reports and white papers) in addition to scholarly databases and search engines (e.g., MEDLINE, Scopus, IEEE Xplore, PubMed, ScienceDirect, Google Scholar) for academic literature.

### 2.2. Study Selection and Charting the Data

The team focused on the latest disruptive trends in the fields of environment and health research, where technologies like IoT, AI, ML, Blockchain, wearable, and ambient environmental sensors were being used to collect and combine data to compare and discover patterns of disease, monitor the spread of diseases, and navigate events that are aggravated by climate change such as heatwaves.

The following search terms were used: “*Artificial Intelligence*”, “*Machine Learning*”, “*Deep Learning*”, “*Blockchain*”, “*Smart Contracts*”, “*Internet of Things*”, “*IoT*”, “*Air quality monitoring*”, “*Air quality*”, “*AAL*”, “*Ambient Assisted Living*”, “*Remote Patient Monitoring*”, “*Climate Change*”, “*Environment*”, “*Monitoring*”, “*Global Warming*”, “*Health*”, “*Healthcare*”, “*Public Health*”, “*Surveillance*”, “*Health Research*”.

Only papers written in English were included in the review. Additionally, only papers that meet at least one of the following criteria were included: It applied any technology we flagged as being of interest in environment or health research, it provided a background view of any of the fields related to this review, or it clarified the challenges of the topics related to this review.

### 2.3. Collating, Summarizing, and Reporting the Results

In the next sections, we present the most recent advances and challenges in the use of the technologies in question for integrating and analyzing environmental and health data. After discussing the disruptive technologies, we present an IoT reference architecture and some uses cases under development by UbiLab team.

## 3. Artificial Intelligence in Environment and Health Research

Governments at the federal and provincial levels often play a central role in monitoring the environment and population health. Many governmental bodies (including environmental regulators) face challenges, such as budgetary and human resource constraints, which can limit the scope of monitoring activities. Applying tools such as AI, ML, and Big Data Analytics can help make essential tasks, such as regulating water pollution levels to protect both the aquatic ecosystems and quality of drinking water, happen in a timely and efficient manner [22]. Computers trained to automatically detect patterns in data, via ML for example, can detect two to seven times as many violations of the U.S. Clean Water Act as current detection methods [7].

While there is no perfect way to manage these considerable challenges, ML can provide a solution. However, researchers stress the importance of recognizing that ML is subjected to algorithm biases. For example, stakeholders may manipulate their reported data to influence their ability to receive benefits or avoid legal penalties. A potential source of bias can happen if the algorithm places more weight on environmental justice concerns by systematically avoiding facilities located in low- or middle-income countries. This approach also fails to consider policy priorities and environmental policies which are not necessarily in a steady state [23].

Possible solutions to these problems include selecting facilities at random, regardless of their risk scores, and retraining the model occasionally to reflect new risk factors. These efforts could help with compliance for low-risk facilities. Building environmental conservation and justice criteria into inspection benchmarks while weighing the positive and negative trade-offs of using self-reported data will be useful to guide best practices in the industry and by individual companies [24].

### 3.1. Machine Learning in Healthcare and Public Health

There are many examples of how ML can be used in public health: image and medical diagnosis; development of warning systems using large datasets like social media where examples might include monitoring for adverse reactions to drugs or users shifting towards suicidal thoughts; predicting hospital readmission; and analyzing genomics datasets [22].

The current Scientific Director of CIHR’s Institute of Population and Public Health, Professor Steven Hoffman, commented on the potential use of AI to address the social determinants of health, such as poverty and housing. These determinants are usually addressed individually, even though they are related to each other, due to the difficulty in comparing necessities. For example, food is expressed in calories or nutrients while income is expressed in dollars. By using ML and AI to create models that consider these relationships, researchers can understand new and complex connections between the social determinants of health, making preventive measures more efficient. For example, professor Hoffman also mentions the use of AI to assist in monitoring and tracking the flu virus [25].

Since public health surveillance involves the monitoring and tracking of population-level data and events to improve the health of populations, aspects that can profoundly benefit from ML techniques are disease surveillance and outbreak. Due to large amounts of data currently being collected from different data sources, including electronic health systems and patient-generated data such as internet searches, connected devices, and social media, techniques involving AI and ML can be very helpful in aiding researchers and public health officials in analyzing and extracting information from data [26].

Currently, researchers have little to no knowledge of global disease distribution. Only 7 (2%) of 355 infectious diseases of clinical significance have been comprehensively mapped for researchers to understand their geography [27]. This may lead to knowledge gaps and seriously impair the ability of public health officials in many countries and worldwide to deploy effective preventive and remedial measures. ML and Big Data approaches can be incredibly useful in identifying patterns and extracting information from geospatial data, and can, therefore, help to mitigate this problem, especially if combined with human oversight and crowdsourcing [27], as seen in the next paragraph.

The potential of ML has been demonstrated in cases that have already used ML in some capacity to help with disease tracking and surveillance. For example, HealthMap provides updated disease occurrence points, using ML to tag and classify if a report or article is breaking news [28]. Other case studies used social-media, such as Twitter, to crowdsource epidemiological data in real-time, using ML to classify the messages’ datasets [29]. Electronic Health Records databases, combined with ML and Big Data, can also be used for public health surveillance [5]. Public health monitoring and surveillance can be improved not only in the context of disease outbreaks but in more mundane health outcomes as well. Several research studies, for instance, use ML to identify injury data written in free text formats [30].

ML is also being used in imaging and medical diagnosis. Researchers from South Korea used ML to identify tumors in images from patients’ exams with 93% accuracy [31]. Google’s computers which are trained to detect breast cancer with AI and ML techniques achieved 89% accuracy compared to 73% for doctors [32].

While ML and AI have been used with considerable accuracy, it is worth noting that reliably measured datasets are required in order for the aforementioned techniques to be valid. For example, a genome sequence is usually perfectly mapped, but administrative data may be lacking in several ways. Related to this problem is the use of secondary data, which are data that are not collected for public health research but are used for it nonetheless, from social media to connected devices’ datasets. Since the investigators were not part of the data collection process, ruling out potential forms of bias becomes more difficult, thus affecting the study’s validity [22,33].

### 3.2. Deep Learning for Modeling Climate Change

As the climate changes due to human activity, it becomes critical to accurately predict weather events that may impact our environment and health. However, climate models often produce very different predictions, primarily because of the way data is broken down into discrete parts, the way processes and systems are paired, and the large variety of spatial and temporal scales [34]. The IPCC reports are based on many climate models and show the range of predictions, which are then averaged out. In averaging them out, this means that each climate model is given equal weight. AI is helping to determine which prediction models are more reliable by adding more weight to those whose predictions eventually prove to be more accurate, and less weight to those which perform poorly. Thus, it is helping to improve the accuracy of both short- and long-term climate change projections.

AI, more precisely deep learning, is also improving weather forecasting and the prediction of extreme, weather-related events. This is because they can incorporate more data and handle the real-world complexity of the climate, accounting for atmospheric and ocean dynamics, and ocean and atmospheric chemistry in their calculations. This improves the precision of weather and climate modeling, making simulations more useful for decision-makers [8].

## 4. Blockchain in Healthcare and Environment

Blockchain is an open-source, distributed ledger equipped with cryptographic techniques that enables trust among parties. The technology is based on the concept of transactions. Today, when a user makes a transaction, it is usually managed by a third-party, such as governments, banks, or insurance companies. The level of security that Blockchain offers is what makes its use so appealing in Healthcare. In Blockchain, when a user makes a transaction in the network, this transaction is validated by the participant nodes, time-stamped, and “sealed” in a block with other transactions. This block is added to a previously existing chain of blocks, hence the name Blockchain [35].

A consensus mechanism adds the transaction among all participant nodes in the network. There are different types of consensus mechanisms. In the case of the Bitcoin Blockchain, consensus is based on a technique called Proof of Work (PoW), which involves a competition between the nodes that maintain the network. Each node tries to guess a random number (called a “nonce”) that solves a mathematical puzzle before every other node. The only way to discover the nonce is by guessing a number and checking if it is the correct answer. Once the nonce is discovered, the winner node uses it as a cryptographic key to seal the block and attach it to the Blockchain [36].

### 4.1. Smart Contracts

The development of the technology and creation of new Blockchains, such as Ethereum, has begun to enable the creation of smart contracts. Smart contracts can be seen as terms of a contract that are written into software, which guarantees the fulfillment of these terms.

While the applications of smart contracts are still in the early stages of development, in the case of healthcare, they can be used in a variety of ways. For example, they can be used to codify the terms of agreement in insurance policies to allow the settlements of health insurance claims in real-time [37]. Another example is to codify consent terms in health studies, which automatically enrolls a participant after their consent is obtained (also removing the patient if the consent is revoked) or assigns a legal caregiver or guardian to a participant if consent needs to be given on behalf of the participant. This example also illustrates how the influence of intermediary third-parties can, in theory, be minimized or removed. The link between smart contracts and IoT technologies can increase automation (or in the cases mentioned above, increase efficiency and patient (or participant) autonomy, while also securing personal data).

Another consideration is whether or not Blockchain is the best solution. In this case, while similar technologies to smart contracts exist, there are several barriers to their adoption such as costs, which are often increased due to intermediaries (for example, the fees from conducting a Bitcoin transaction were estimated to be 5.5 times lower than fees from a credit card in 2016) [35].

### 4.2. Blockchain in Healthcare

Healthcare is a complex field with a lot of different stakeholders, such as patients, doctors, hospitals/clinics, researchers, insurance payers, and pharmaceutical companies, all of which operate on overwhelming amounts of patient data. The field is increasingly digitalized, which can provide growth opportunities in areas such as personalized medicine, improved health services, and better care. For example, Electronic Medical Records (EMRs) that digitally store a patient’s health information were introduced in the 1970s to improve billing and are increasingly being used by stakeholders [38].

Since the providers are the primary stewards of the data, and their systems suffer from data sharing and interoperability issues with other providers, patients lose access to their past health data, thereby impeding a holistic view of health. The process to access the full record is challenging. For example, providers in the U.S. can take up to 60 days to respond, and not necessarily comply, to requests for updating an erroneously added request [39]. Most health data are subjected to strict regulations, varying across geographical regions, pointing to an increasing need for compliance and auditability. Ultimately, the issues faced by this system can lead to abuses by health companies as well as poorer health services and outcomes [39].

Besides siloed patient data, security is another concern. The Office of Civil Rights of the U.S. Department of Health and Human Services reported that more than 15.5 million EMRs were breached in the U.S. in 2016, and, in the same year, it is estimated that the healthcare industry spent $6.2 billion to deal with healthcare data breaches. The estimated value of an EMR is approximately $500 on the black market, which is 10 times more valuable than a credit card number [40].

In addition to problems with patient data, other challenges are affecting the current healthcare landscape. In the health insurance context, there is a lack of trust and connectivity between payers, providers, patients, and healthcare companies, which prevents coordination of care and leads to an increase in costs. The genomics research field also suffers from costs and trust issues, generating barriers for the creation of large genomic datasets that are needed for research [41,42]. In the drug supply chain scenario, in 2017, it was estimated that an annual 200 billion USD is lost to counterfeit drugs, and a unified tracking system among manufacturers that provides end-to-end traceability does not exist [10,43].

While there is no single solution to all the problems in the industry, many experts and industry stakeholders are identifying the benefits of applying Blockchain technology in the field. IBM released two reports in 2016 and 2018 surveying more than 400 healthcare and life sciences executives from 18 countries on the use of Blockchain. 56% of healthcare executives and 70% of life sciences executives had plans to adopt it by 2020 [44,45]. In 2018, the Blockchain-healthcare industry’s value was estimated to be around 175 million USD, and this number is expected to grow to a billion USD in 2025 [46].

Experts believe that Blockchain can help improve issues in the field such as trust, transparency, data fragmentation, data sharing, and interoperability. Access and sharing of information between different entities and/or databases, such as Health Canada and provincial ministries of health, can be facilitated using Blockchain. In terms of sharing EMRs between hospitals, Blockchain can provide a common infrastructure for the authorization and sharing of information where the references to medical data from different sources would be stored on the Blockchain, creating a breadcrumb trail of medical history. It is important to note that the actual personal data would not be stored on the Blockchain. Firstly, Blockchains are not to be used as databases; because of the distributed and consensus mechanisms, they are rather inefficient at storing data and should be used as transparent and tamper proof ledgers. In addition, current Blockchain implementations do not allow for deletion of personal data, which users and patients are legally allowed to do under legislative acts. In the case of EMRs, only the references to where personal data are stored are recorded on the Blockchain, not the data itself [45].

Blockchain enabled solutions can increase patient control and provide patients with access to their connected records, coupled with the ability to choose with whom they wish to share their data. Solutions such as MedRec [39] and HealthBank [47] are developing such EMR solutions. An EMR solution could potentially be improved with Blockchain-enabled platforms to manage consent in health research and treatment. In this scenario, Blockchain can provide an immutable and timestamped log of consent and increase data sharing, transparency, and the safety of stakeholders. IBM is developing a solution with Blockchain for clinical trials to manage patient consent, and the UbiLab is also currently researching the application of Blockchain solutions that expand this scenario through allowing consent for different data types by guardians or caregivers of patients.

It is important to note that while such solutions may empower patients to own their own data, they can also raise security and privacy issues. In the EMR context, for example, the references to all the data will be stored in one place. Most Blockchain applications are currently in the initial implementation or concept stages, and these issues will need to be addressed, such as by considering the implementation of a consortium Blockchain or different consensus mechanisms, in order to create safe, efficient, and reliable solutions. There are also limitations in Blockchain that specialists are experimenting on and doing research to solve, such as scalability issues. Nevertheless, Blockchain technology is a promising approach for improving on several issues that affect the healthcare field.

### 4.3. Blockchain in Environment

Much like in healthcare, Blockchain can help solve interoperability and data sharing issues, leading to better information for decision-making and optimization of resources. Pressing environmental concerns, such as energy, climate change, natural disasters, ocean-health deterioration, and air pollution, amongst others, can also be improved with the technology. For instance, we can cite an analogous case of drug supply chain tracking with food products. A Blockchain-enabled solution to track food can increase transparency on the entire manufacturing process and enable customers to make environmentally friendly decisions. Companies such as IBM are already in the early stages of implementing food supply chain solutions powered by Blockchain. The tracking of fish provenance or endangered species is another potential application [48,49].

Much has been said about Blockchain’s potential to enable peer-to-peer trading. In environmental scenarios, this could initiate the creation of peer-to-peer energy-trading systems, where users could trade energy between themselves. It could also be a disruptive technology in places affected by power outages and optimize the entire energy market. The technology could create new markets and increase the value of resources that are currently wasted. There are initiatives to encourage participation in recycling programs and activities by rewarding users who deposit recyclables with cryptographic tokens that can be exchanged for other goods or services. In the same area, a Blockchain-based tracking system for carbon use, with reputation and reward mechanisms, could encourage people and companies to minimize their carbon footprint and improve auditability and regulation compliance with environmental policies and treaties [50].

The integration of Blockchain with IoT sensors can lead to more transparent, efficient, and secure monitoring of variables such as air and water pollution, and Blockchain’s resilience can help with natural disasters [24]. These are just some of the proposed applications of Blockchain within the environmental sector. It is important to note that Blockchain by itself does not form a complete solution in most of these cases by depending only on technologies such as IoT and AI. For automated energy markets, for example, smart meters and other sensors are necessary. The use of IoT can also improve one of the challenges embedded in Blockchain. Despite providing an immutable log of data, the input of these data must be reliable and accurate. Automated inputs with the use of IoT devices minimize errors and increase the reliability of data. In this case, however, it is essential to be aware of the need for security with regards to IoT devices as they may be vulnerable to hacking.

Besides the problem with guaranteeing accurate inputs, some critics of the technology argue that Blockchain and cryptocurrency, in general, expend a tremendous amount of energy in computations, and by definition are not environmentally friendly. However, Blockchain is still maturing, and new techniques and consensus mechanisms can help in diminishing the carbon footprint of the technology. Most experts recognize the benefits and untapped potential that Blockchain can bring to the environmental sector, especially if combined with IoT, AI, and other innovative technologies [51].

## 5. Internet of Things in Environment and Health

As described by Ziegler [52], IoT plays a growing role in varying domains, including environmental monitoring and health. This section describes current uses of IoT technology within these domains, providing context for some of the potential data sources (Ambient Assisted Living—AAL, Remote Patient Monitoring—RPM, Environmental monitoring, and Public Health Surveillance) that could be considered in scoping and developing the pan-Canadian surveillance system.

### 5.1. Ambient Assisted Living—AAL

Aging populations continue to be a major concern for governments and health care systems around the world [53]. To support independent living for aging populations, researchers and innovators have been exploring the use of wearables, sensors, and mobile health technologies to improve their quality of life [54].

One possible solution that promotes independent living and aims to allow aging or other vulnerable populations to maintain a high quality of life is the use of ambient assisted living (AAL) technology. According to the International Electrotechnical Commission/Systems Committees Active Assisted Living (IEC SyC AAL) [55], the use of AAL support systems for elderly people in industrialized countries has been shown to increase general health and quality of life measures. An example of how these technologies can support this population is a smart thermometer that automatically cools the house once it detects a heatwave, thus preventing the elderly from experiencing heatstroke. Technology is a vital component of offering a higher quality of life for an aging society or for anyone who needs extra help performing their daily living activities inside or outside their homes [56].

The International Medical Informatics Association (IMIA) approved the creation of a new working group on smart homes and AAL [57] in November 2006. The AAL ecosystem can integrate assistive technologies, smart homes, and telehealth services, multi-sensors (for gathering data and monitoring individuals in their homes), individual health/wellness trackers, and homecare-based ambient trackers [58]. The goal of Ambient Intelligence (AmI), is to assist users during everyday activities by being embedded in home environments [59].

AmI applications are transparent and invisible for users, while security and privacy requirements are guaranteed [60]. With dementia and Alzheimer’s disease as growing concerns in Canada [61], there is more emphasis on the use of technology to improve the overall quality of life for patients living with these conditions [62].

While Canada is not alone in facing a growing aging population [61], Canada lags compared to other countries in the use of AAL technology [58]. Countries in Europe and Asia are at the forefront of developing and deploying large-scale platforms such as the Inclusion Society project [12], a project of the AAL Programme. Europe has the most advanced programs for leveraging AAL technology in healthcare [58], while Japan, with the world’s oldest population and research focused on the creation of robots to support their aging population, is one of the most advanced countries in the development and adoption of AAL technologies [63].

### 5.2. Remote Patient Monitoring—RPM

The expenditure on healthcare in Canada is increasing due to chronic diseases and the aging population. One approach to dealing with the issue is to monitor patients outside of hospital settings [64]. Remote Patient Monitoring (RPM) is defined as “the delivery of healthcare to patients outside conventional settings enabled by a technological application or device. It hinges on the electric transmission of patient data to a provider as a series of integrated services and processes, ranging from health coaching to the alteration of a patient’s course of care” [65].

Using telehealth, mHealth, wearable, and IoT technologies, RPM enables care providers to continuously monitor and deliver care to patients regardless of their geographical location. For example, they can detect when a patient is suffering from an asthma attack due to a sudden increase in air pollution and alert the closest emergency services in real-time to provide immediate relief. Continuous monitoring detects adverse health events early and reduces their risks, improves monitoring of medication compliance, and reduces unnecessary and/or inappropriate treatments [64]. Thus, RPM could potentially reduce patients’ needs for more complex interventions [65], which in turn can reduce the number of emergency department visits and hospitalizations, caregiver burden, and health care costs [66].

Since patients can send their collected data to care providers whenever and wherever, RPM can provide patients with their necessary care in outpatient environments, such as their homes [67], thereby reducing the number of in-patient visits [68], transportation costs [64], waiting times for services, and the costs of personnel and administrative operations [68]. This also increases the efficiency of health resource allocation [67] and convenience for patients, which can improve their quality of life as they could spend their time enjoying other activities instead of seeking out health services [68].

By providing continuous, real-time data to care providers, RPM technologies have the potential to improve patient safety, as well as the quality of and level of access to healthcare, which could then improve the cost management of health resources [64]. However, some of the challenges of using IoT technologies in RPM are the concerns regarding the accuracy and reliability of the data captured and measured by consumer-level technologies [69]. Additionally, most commercial technologies are not designed to support data collaboration between patients and care providers during clinical visits and beyond [70].

### 5.3. Environment Monitoring

Extreme weather-related events due to a changing climate, such as dramatic changes in temperature, forest fires, and rainfall levels which lead to flooding, have a direct impact on health. For example, the increasing frequency of heatwaves, especially in urban areas, will increase the incidence of heat-related health issues such as mortality, especially amongst the elderly. People are dependent on their ecosystem, and their health is directly affected by these changes, including the increased distribution and transmission of disease and air pollutants [71].

Monitoring environment and health through IoT could help improve current understandings of their interaction, leading to potential solutions to mitigate negative consequences. Current uses of IoT devices in the environment include mapping spatio-temporal data, such as transportation rates and sources of pollution, and using the data collected from sensors and/or crowd-sourced from users for epidemiological monitoring [72].

Air quality monitoring sensors are mostly used for outdoor and/or indoor monitoring [73,74,75,76] and personal monitoring [77,78]. Wearable sensors can provide estimates of personal exposures to different types of pollution, and, with the use of the Global Positioning System (GPS), this data could be used to estimate the spatial distribution of air pollutants in different micro-environments [72].

Aside from air pollution, IoT technologies can also monitor and produce real-time maps of water pollution, noise levels, temperature, and harmful radiation [79]. For water monitoring, sensors that detect water levels could provide early warnings of water disasters, such as floods, allowing people the time to evacuate or plan in advance of the event [80]. Similarly, radiological risk monitoring helps reduce or eliminate the consequences of the release of radiation elements by providing early detection [81]. Sensors that could predict air temperature and relative air humidity could also help detect and manage the spread of fungal diseases in crop fields, which are important for providing food to the population [82].

When these IoT products are used with crowd-sourced maps, they could further support crisis management by monitoring crises, such as earthquakes, floods, and radiation levels; coordinating disaster relief efforts; providing support from outside disaster zones; and keeping users informed and aware of the crises [83]. Even though these maps are not IoT products alone, their usage with sensors that monitor factors in real-time could form systems that could additionally provide early detection and warnings of natural disasters [84].

Some of the challenges of using IoT technologies in environmental applications are that there are, again, concerns regarding the accuracy and reliability of the data captured and measured by consumer-level technologies [69]. There are also concerns with the accuracy and quality of the data provided by users since they might potentially add their biases to the shared data, share “popular” but incorrect data, or collect and share useless data due to being inexperienced or uninformed in their understanding of data collection and use [85]. The biases and misinformation contained in raw data mined from social networks could be especially problematic for crowdsourced maps as they rely on user-provided data [83].

### 5.4. Public Health Surveillance

In Public Health, surveillance is defined by the World Health Organization as “the continuous, systematic collection, analysis, and interpretation of health-related data needed for the planning, implementation, and evaluation of public health practice” [86]. Surveillance is essential in the development and deployment of prevention and control measures by public health agencies. Current technological advancements allow data collection and analysis on an unprecedented scale that requires improved surveillance methodologies [87].

Society is moving into an age of smart technologies that monitor health, such as mobile and wearable products, IoT solutions, and ambient assisted living (AAL) systems including smartphones, smartwatches, wireless scales, wireless blood pressure cuffs, and smart thermostats amongst others. For example, ecobee, a smart Wi-Fi thermostat company, have an existing user base of tens of thousands of thermostats around the globe with embedded sensors that allow for surveillance and real-time collection/analyses of health related data [88].

Some related work focuses on the usage of IoT technology for supporting health surveillance. For example, mPower is an app that leverages Apple’s health data collection framework HealthKit (centralizing data from different sources like smartphones and smartwatches) and uses the iPhone’s gyroscope to measure balance, dexterity, and memory to understand Parkinson’s disease better [89]. The app has enrolled over 10,000 participants (93% of them never took part in any study) and became the largest study on Parkinson’s in history [90].

Another exciting way which IoT and related technologies can change society are smart cities, which are IoT-powered cities aimed at “improving the quality of life of their populations in a variety of ways, including through measures that promote eco-friendly, sustainable environments and delivery of connected health/care’ services to citizens” [91]. The Spanish city of Barcelona, for example, partnered with Cisco in the application of IoT devices and systems throughout the city, and consequently enhanced their services. Barcelona’s bus stops display real-time bus timetables based on these devices and the city’s parking spots detect the presence of cars and also allow for online booking and payments [91]. The city of Waterloo, Canada, teamed up with industry partners to set up online wireless sensors that detect leaks in the city’s waterlines, enabling faster failure detection as well as the deployment of preventive measures [92]. As shown in the examples, surveillance via smart cities can help manage various environmental issues and ultimately improve a city’s environmental management and sustainability [91].

There are several ongoing concerns in public health surveillance that need to be overcome. These issues can be technical or non-technical concerns related to data use and access. The lack of infrastructure, skilled human resources, and adequate funding are the main challenges for public health surveillance, and this remains predominantly unprioritized. Non-technical issues include ethics, privacy, and security concerns, but obtaining informed consent is considered crucial for the application of research in healthcare [93]. For example, consent needs to be sought from caregivers, legal guardians, and others who may have their data collected for surveillance involving interconnected devices, as in the context of the older populations and using IoT devices to support independent living and while monitoring their safety. This is also related to more pressing issues of privacy and data security, which applies not only to health studies and monitoring but to surveillance in general. For smart cities, for example, a person’s security and privacy needs to be preserved. Data-related challenges concern data use, access, database management, storage and analysis. In addition, difficulty in interoperability between data systems, lack of standardization of data, and lack of protocols to validate data reliability and trustworthiness are critical concerns.

Current issues that hinder the development of unified systems for data access and use are, among others: health data siloed and stored by different providers, each with its own system that is not interoperable with other systems, which greatly increases the difficulty of data sharing between different stakeholders [39,47]. Ultimately, this affects data generation, access and use, and is troublesome considering that new technological advances, like Big Data, need large datasets for application. As an example, large datasets of genomic data are needed for research in the field of genomics, but high costs, data sharing/interoperability issues, and privacy concerns stop patients from sequencing their genomes, affecting the availability of genomic data [94].

#### Challenges in the Use of IoT Technology for Surveillance

Development and implementation of IoT lacks the coordination and trusted curation needed to ensure efficient identification of best global practices, harmonization, and standardization. Without a coordinated effort to identify best practices and share them with all nations, countries would be left to independently assess and experiment with methods in order to incorporate the new technologies into their national surveillance programs [95]. Technological advancements increase the availability of datasets in different contexts (e.g., clinical, environmental, and insurance). To identify patterns and correlations among these different data types, it is necessary to organize the data into standard formats that can be used for public health officials, statisticians, and other stakeholders. This exacerbates the problem because many systems were not developed with interoperability in mind and those systems are subjected to laws and regulations that, many times, cover distinct data collection, storage, and sharing rules as well as different views on security and privacy [96].

## 6. Software Architecture for the Pan-Canadian Monitoring and Surveillance Activities Related to Environment and Health

There is no doubt that the reality of a world full of interconnected devices, with great potential to support healthy living while acting on behalf of individuals, is close at hand. From a software engineering perspective, for this scenario to become a reality, it is necessary to first address several technological challenges through the careful selection of a proper software architecture for surveillance systems [97]. Due to the similarity of the challenges in the scenarios presented above, it is possible to conceive of a general architecture that addresses most of the requirements and that will enable the development of potential projects using data from the pan-Canadian surveillance system.

**Figure 1:**
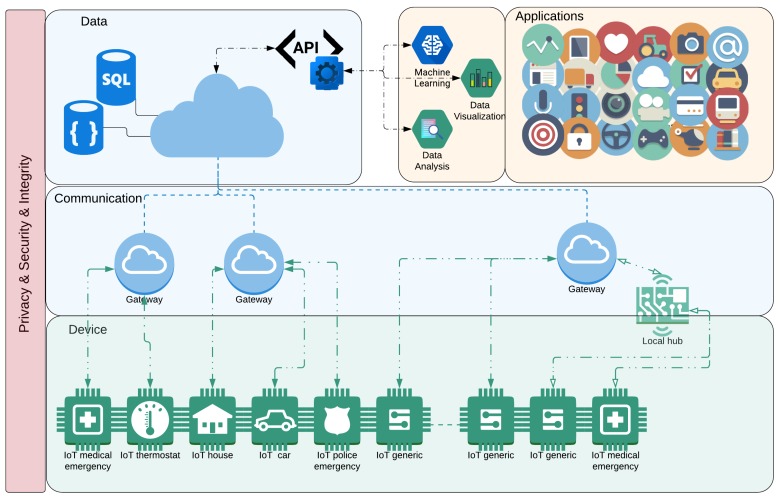
A Recommendation for a General Architecture for the pan-Canadian surveillance system proposed by UbiLab. This architecture is composed of the following main layers: (i) **Device** representing the different types of (IoT) devices that can be integrated into the system, (ii) **Communication** enabling the devices to communicate with the data sources on the Internet, (iii) **Data** representing and storing the information generated and collected by the devices, (iv) **Applications** representing the consumers/providers of information, and (v) **Privacy and Security** a cross-layer to contemplate the triad of confidentiality, integrity, and availability.

Figure 1 shows the conception of a proposed general architecture that could be considered for the pan-Canadian surveillance system. The main objectives and potential use cases of the system’s components were also taken into account when conceiving of the architecture. The main components/layers of this architecture are Device, Network, Data, Applications, and Privacy, Security, and Integrity, each described in the section below.

### 6.1. Device and Communication

The device layer represents the different types of devices that can be integrated into the system. In this case, it is salient to highlight the central concept associated with the IoT devices and fundamental for the proposed architecture: Internet access [9]. That said, a device can be (or can act like) a sensor, or an aggregator of a sensor (for instance, a thermostat device can have a temperature sensor, as well as a humidity sensor), or an actuator. In general, these devices have restrictions regarding their capacity for processing power, memory capabilities, and batteries [98]. These devices could also be something more powerful, such as within a car or within a smart home (which can aggregate a series of devices or hubs), and how it will be classified depends on how the information is going to be used/consumed by applications [99].

One essential aspect of the devices is that they need to have access to the information from environment. It is very common, mainly when we are talking about sensors, that they need some auxiliary device to have proper access to the information. For example, a smartwatch can communicate with the internet by using a smartphone as a gateway, or it can even be used for running edge computing services [100].

Once the devices start to collect (or generate) information, it is necessary to make the data available for other devices or applications. That is the role of the Communication layer, to enable the devices to communicate with the data sources on the Internet [101]. To accomplish this level of communication, various protocols and technologies can be used. Some of the protocols currently available or are planned for soon are: Wi-Fi, 2G/3G/4G/5G, Metro Networks, PLC, xDSL, and FTTx [57]. Notice that in this layer, we are not concerned with the M2M communication or with the connection between the devices and the hub [101].

### 6.2. Data and DataAPI

The data layer is responsible for representing and storing the information generated and collected by the devices. These devices will generate vast amounts of data which need to be stored, managed, and analyzed by Big Data Analytics [102]. For a better description of the complexity of this layer, we present the most common components associated with Big Data processing architectures (for example, the layers inside our Data layer) such as Data Ingestion, Data Collection, Data Processing, Data Storage, and Data Query [103].

One important feature to be addressed in the Data Layer is the capacity for making Data Analysis and ML processes easier [104]. This feature is addressed by the DataAPI, which is a service provider that pre-processes the data to give a better flow for Data Analysis [105]. This includes: the background services for identifying and cleaning anomalous and dirty data; the services for adding security and privacy aspects to the data, such as the encryption necessary for sensitive data, or even an additional authentication and access control service; or even services that can processes the data for specific purposes, as could be the case for the pan-Canadian surveillance system.

### 6.3. Applications

Here is where we can benefit the most from the IoT architecture/infrastructure. In this layer, we have applications that are the consumers of all the information that has been collected. Considering the scope of the application, the ten most commonly used IoT applications, according to Lueth [106] are Smart home, Wearables, Smart city, Smart grids, Industrial internet, Connected car, Connected Health (Digital health/Telehealth/Telemedicine), Smart retail, Smart supply chain, and Smart farming. With regards to connected health, the author states that it “...remains the sleeping giant of the IoT applications. The concept of a connected health care system and smart medical devices bears enormous potential... not just for companies but also for the well-being of people in general. Yet, Connected Health has not reached the masses yet. Prominent use cases and large-scale startup successes are still to be seen” [106].

Notice that these applications are usually executed on the Web and on the devices that were described in the Device layer. The execution of ML algorithms and Data Analysis would be treated as applications on this ecosystem [107]. In this case, these applications are not necessarily associated with a device as they can be performed directly on the cloud. Regarding data access, the application uses the provided DataAPI, that provides the data with some pre-processing for assured security and privacy of data, as well as to have the data adjusted to the application domain. It is worth noting that if an application or ML algorithm needs to access the data as they are (without pre-processing), this can be done since the application has authorization.

### 6.4. Security and Privacy

Security and privacy are amongst the most concerning aspects of the IoT, in ethical, legal, and technological terms. For security, it is important to contemplate the triad of confidentiality, integrity, and availability [108].

It is not unusual that the data handled by IoT applications are confidential. To ensure that these data, classified as confidential, will not be misused, it is possible to use encryption methods. However, it is necessary to properly analyze the effectiveness of these methods in the context of IoT, considering mainly the aspects of applicability and criticality of the IoT applications. This is important because wireless communications may be more susceptible to hackers’ attacks that can compromise the confidentiality of communication. IoT integrity refers mainly to physical failures and damages, at first sight, integrity protection includes preservation against sabotage and the use of counterfeit units or components [109].

One way to achieve the desired integrity is by using authentication methods. This tries to guarantee the identity of a particular user or object and also focuses on assuring its integrity. Authentication, through Identity Management (IdM), provides resource control and helps to also deliver auditing, accounting and access control [108].

The availability requirements are very close to reliability. In this sense, it is critical that the IoT infrastructure addresses some security issues to avoid (or prevent) attacks that can make the service unavailable, such as, Distributed Denial-of-Service (DDoS). This is another reason why Blockchain emerges as a good solution since it uses a P2P network, which is decentralized, without a single point of failure, it is more resilient than centralized systems.

## 7. Use Cases

In this section we present some use cases to frame potential scenarios in which the technologies discussed above could be applied for collecting data, analyzing and predicting outcomes with the purpose of better understanding or improving the health of Canadians.

### 7.1. Automated Environmental Control

As stated in the Blockchain section, technology’s features can be leveraged to implement smart contracts with terms coded into software that guarantees the fulfillment of these terms (“digital promises”). These contracts can be automated to provide new products and services in several fields [110].

A Gartner report describes, in the health insurance area, a theoretical scenario in which a smart contract is implemented and combined with crop sensors. If there is any damage to the crops, the contract can manage an automatic claims settlement for the damage. Receiving the settlement might be the first time that the crop’s farmer will be aware of the damage [111].

This automation can significantly benefit the environment and healthcare area. Smart contracts can be developed to receive inputs from IoT sensors that measure environmental and behavioural variables such as movements in the house, indoor or outdoor temperature, and energy use, as well as the water level in the household or the public system, in addition to many other variables. If a variable is achieving dangerous threshold levels, the contracts will be triggered to activate preventive measures, such as enabling other integrated devices (e.g., activating smart thermostats located around the house to lower indoor temperatures) and giving automated warnings.

Such a system could potentially be combined with aggregated data from a geographical area and combined with new technologies, such as Big Data and Machine Learning, to identify patterns and act. For example, if an extreme heatwave is being detected in a Canadian province, the contract can be triggered to lower household indoor temperatures in the area and prevent heat strokes.

Besides, smart contracts can also be used to automate the storage and sharing of environmental and health data. Once it receives input from the IoT sensors, the contracts can have hardcoded rules to directly share this data, in a cryptographically secure way, with researchers or public health and surveillance agencies using Blockchain.

It is important to note that for such a system to be implemented, under Canada’s privacy regulations such as the Personal Information Protection and Electronic Documents Act (PIPEDA) [112]—which set parameters for the management of personal data by commercial activities—obtaining consent for the collection, use and disclosure of user data is necessary. In addition, there are security concerns that need to be taken into account during the development process, such as the hacking of the devices—which may lead to data leakage and to inaccurate information being sent to the Blockchain—and the question of how to model the information flow in the system—if a public Blockchain is used, for example, the data will be available to all those who wish to see it; this may not be a problem for information collected from public areas, but it may violate privacy concerns in private residences.

### 7.2. Monitoring Air Pollution at Schools

Air pollution is responsible for millions of premature deaths and results in diseases and developmental issues among pregnant women, and consequently children who are exposed to pollutants in the womb, newborns, and preschool children around the world. State of the art air quality monitoring, and data collection and management systems are needed to understand the impact air pollution has on maternal and child health. Due to this lack of understanding, Family Health Centres (FHCs) and hospitals are overwhelmed and unprepared to adequately treat air pollution-related diseases such as pneumonia.

Part of the action plan involves improving the ability to generate and disseminate data, research, analysis, and information on air pollution and maternal and child health. In this context, it is possible to conceive of a Big Data infrastructure where outdoor and indoor air pollution data will be collected from air quality monitoring devices spread across districts. This enables the development of an online platform for real-time air quality monitoring as well as the development of a mobile app for the same purpose. The idea is to collect data about particulate matter (PM2.5 and PM10) from the air quality monitoring units. Additionally, other types of data can also be collected for further analysis, such as CO2, TVOC, temperature, humidity, and information about wind from outdoor measurements. This platform will link the air quality monitoring data with child health data gathered from different sources such as, emergency medical records (EMR) or other health data, which will be used to create algorithms that can potentially predict maternal and child health outcomes related to air pollution. This will further educate healthcare practitioners and better prepare FHCs and hospitals to address the air pollution-induced maternal and child health crisis.

The online platform and mobile app will be used for the knowledge management and knowledge exchange of air pollution monitoring, and maternal and child health data. This platform will allow healthcare practitioners to access up to date and reliable data which will provide them with the knowledge they need to deal with the short-, medium-, and long-term public health consequences of air pollution. Besides, reliable air quality monitoring, the maternal and child health data generated from this platform can be used to inform policy formulation, health care reforms, and develop educational material.

### 7.3. Look for Changes in Diseases and Death Pattern with Time

As most developing countries around the world have transitioned out of the infectious disease phase, they have shifted into the phase of chronic disease. All G7 countries have seen a decrease in mortality, and forecasting the dependency rations (ration of people over 65 to working people) is important for governmental planning [113].

The incidence of chronic diseases has risen with economic development in the last 50 years, such as cardiovascular disease and the family of nutritional risk factor diseases (overweight, obesity, cholesterol and elevated blood pressure) are the leading causes of global mortality and morbidity [114]. Using IoT sensors to detect and measure disease symptoms in real-time as well as AI to track and map diseases, it is possible to learn insights about how the patterns of disease have changed over time. Along with this, the death patterns have also shifted. Knowing how these patterns change with time will allow more efficient allocation of health resources to better improve population health.

One popular method called public syndromic surveillance used extensively in the United States of America, uses data from inpatient and ambulatory clinics. Public health departments at the state and local levels can deal with disease outbreaks and quickly respond in emergencies with timely access to EHR data [115]. ICD codes (current and previous versions) are used to map the causes of death and observe disease trends and pattern changes across geographical locations over time.

With the advent of social media, it is also possible to scrape the internet for keywords and pictures and analyze them to predict trends. Are people frequenting a certain spot, and is there increased traffic, potential risk of personal injury, or potential harm to the environment? This could be used to monitor visitors of National Parks, for example. Are people taking pictures in a dangerous location or engaging in unsafe behaviours? Could the increase in human traffic in a particular area of the park be causing undue harm to the environment or wildlife? Or there could be a music festival, and there is an increase of people reporting that they are experiencing heat exhaustion due to high temperatures. By knowing these trends, timely preventive measures could be implemented. National Parks could build paths that redirect people away from dangerous locations and disperse the traffic to levels that no longer harms the environment. A smart thermostat could automatically cool a venue when an increasing number of people are experiencing heat exhaustion. The possibilities of linking social media to environment and health data are endless.

## 8. Discussion and Challenges

Much of the literature reviewed echoed statements around the need for transparency before the successful adoption of a data shared infrastructure could happen because ambiguity regarding the handling of private data causes a lack of trust. In previous research conducted by the UbiLab related to AAL technology, and for this project, several challenges pertaining to the implementation of health surveillance technology emerged. The main challenges were: interoperability; data-sharing and data-governance; as well as privacy, trust, and security. These challenges are presented below, highlighting some of the areas that Health Canada may wish to consider in the scoping and development of the pan-Canadian surveillance system.

Interoperability between technologies is critical for the successful deployment of a system-of-systems integrating existing datasets, mHealth, and any IoT technology. Devices may be interoperable from a technical point of view, but manufacturers may opt to keep their data within their platform to guard their proprietary algorithms and make sure other competitors do not use their data. Some standards and protocols, such as HL7/FHIR, work to ensure that the data is exchanged in a format that is structured and comprehensible for both parties. Unfortunately, the issues are not just in the communication itself but the difficulty of data exchange between systems, data incompatibility, and data governance [116].

Since data are collected from a variety of sources, the creation of data sharing policies should involve multiple stakeholders [117]. This heterogeneity makes it difficult to determine data ownership and data governance. For example, it is challenging to understand when the data no longer belongs to the individual and their device (e.g., smartphone or smart hub) but to a company, government, or healthcare institution instead, where it becomes medical information. Additionally, data sharing also faces obstacles related to the availability, reliability, integrity, validity, and accuracy of the data collected. The distribution of data collected through IoT devices for healthcare applications must consider the ownership and control of access to family members or caregivers, revealing issues of security and privacy.

In a related vein, a major concern regarding data sharing is transparency and trust. The result of a survey presented by TrustArc in 2014 regarding consumer attitudes towards data collection through smart devices showed that: 85% want to understand more about data being collected; 88% want to control the data being collected; 83% are concerned about the idea of information collected; and 87% are concerned about the type of information collected. Without the foundation of proper trust, the technology cannot deliver all the benefits offered to its users [118].

The end user needs to be the owner of all their data and they should have the right to know what is being collected, who will use the data, how that data will be used, and the benefits of collecting their data. It is necessary for vendors to improve the way they communicate their use of data and assume responsibility for transparency in order to achieve this goal. It is also necessary to raise awareness about the importance of having control and knowledge of one’s data with the option for users to opt out at any time and choose with whom they prefer to share their data and for which purposes [119].

The use of IoT devices in health applications has an even greater impact on privacy because the exchange of personal information is not yet precisely regulated in these technologies [120]. Issues such as equity also cause concerns about the fact that only middle and high-income individuals benefit from the implementation of a system using high-end IoT devices to improve their daily life. This issue is closely tied to the need for greater transparency and clarity of data ownership and governance. Canadians are concerned about their privacy, lack of trust, and fear the ramifications of sharing their health data. The public seeks the benefits of data sharing and personalized services, but their negative perception is shaped in part by mainstream media that portrays the future of technology as a dystopian landscape where the individual loses control of their digital footprint. People fear that their data may be used inappropriately, leading to undesirable consequences, such as having insurance companies deny them coverage for certain services after gaining access to their health data through a third party.

Technology has been evolving at an exponential rate. As we move to adopt more sustainable and transparent practices, it is imperative to build a suitable infrastructure that supports technologies that have the potential to be incorporated into the pan-Canadian surveillance system. In this sense, the proposed reference architecture monitoring and surveillance can be used to develop successful use cases.

## 9. Conclusions

To scope and develop monitoring and surveillance activities related to environmental impacts on health and the health system, the Ubiquitous Health Technology Lab (UbiLab) at the University of Waterloo proposes to engage multidisciplinary stakeholders, which could include government, public researchers, various industries, service providers and/or innovators. Previous projects conducted by UbiLab highlight that policy gaps regarding data governance, privacy, and security should be addressed before successful surveillance activities are implemented. The general population, especially the younger generation, is open to the idea of a technology-driven and technology-permeated lifestyle but there is still an issue of trust and a need to educate and develop digital literacy skills.

While technologies like IoT, Blockchain, and AI have great potential to support initiatives integrating health and environmental data, including potential to be part of a pan-Canadian surveillance system, there are some limitations and challenges related to the use of these technologies in health surveillance that should be addressed. This paper provided an overview of current examples in the use of these three technologies, focusing on surveillance and remote monitoring, and presented a proposed software architecture in which these technologies could be combined in the future in the form of pilot studies and surveillance activities. The intense work of reviewing numerous research papers and government reports and the proposal of the technological architecture for monitoring and activities provides interesting information for future research.
